# Diamine Oxidase-Conjugated Multiwalled Carbon Nanotubes to Facilitate Electrode Surface Homogeneity

**DOI:** 10.3390/s22020675

**Published:** 2022-01-16

**Authors:** M. Amin, B. M. Abdullah, S. J. Rowley-Neale, S. Wylie, A. J. Slate, C. E. Banks, K. A. Whitehead

**Affiliations:** 1 Department of Engineering and Technology, Liverpool John Moore’s University, Liverpool L3 3AF, UK; B.M.Abdullah@ljmu.ac.uk (B.M.A.); s.r.wylie@ljmu.ac.uk (S.W.); 2 Microbiology at Interfaces Group, Manchester Metropolitan University, Manchester M1 5GD, UK; 3 Faculty of Science and Engineering, Manchester Metropolitan University, Manchester M1 5GD, UK; s.rowley-neale@mmu.ac.uk (S.J.R.-N.); c.banks@mmu.ac.uk (C.E.B.); 4 Department of Biology and Biochemistry, University of Bath, Claverton Down, Bath BA2 7AY, UK; ajs319@bath.ac.uk

**Keywords:** biosensors, covalent conjugation, enzyme, electrochemistry, surface characterisation

## Abstract

Carbon nanomaterials have gained significant interest over recent years in the field of electrochemistry, and they may be limited in their use due to issues with their difficulty in dispersion. Enzymes are prime components for detecting biological molecules and enabling electrochemical interactions, but they may also enhance multiwalled carbon nanotube (MWCNT) dispersion. This study evaluated a MWCNT and diamine oxidase enzyme (DAO)-functionalised screen-printed electrode (SPE) to demonstrate improved methods of MWCNT functionalisation and dispersion. MWCNT morphology and dispersion was determined using UV-Vis spectroscopy (UV-Vis) and scanning electron microscopy (SEM). Carboxyl groups were introduced onto the MWCNT surfaces using acid etching. MWCNT functionalisation was carried out using 1-ethyl-3-(3-dimethylaminopropyl)carbodiimide hydrochloride (EDC) and *N*-Hydroxysuccinimide (NHS), followed by DAO conjugation and glutaraldehyde (GA) crosslinking. Modified C-MWNCT/EDC-NHS/DAO/GA was drop cast onto SPEs. Modified and unmodified electrodes after MWCNT functionalisation were characterised using optical profilometry (roughness), water contact angle measurements (wettability), Raman spectroscopy and energy dispersive X-ray spectroscopy (EDX) (vibrational modes and elemental composition, respectively). The results demonstrated that the addition of the DAO improved MWCNT homogenous dispersion and the solution demonstrated enhanced stability which remained over two days. Drop casting of C-MWCNT/EDC-NHS/DAO/GA onto carbon screen-printed electrodes increased the surface roughness and wettability. UV-Vis, SEM, Raman and EDX analysis determined the presence of carboxylated MWCNT variants from their non-carboxylated counterparts. Electrochemical analysis demonstrated an efficient electron transfer rate process and a diffusion-controlled redox process. The modification of such electrodes may be utilised for the development of biosensors which could be utilised to support a range of healthcare related fields.

## 1. Introduction

The use of electrochemical analysis remains a cost effective and simple method to determine the concentration of electroactive species in solution [1]. The morphology, size of the electrode and the fabrication method utilised play a major role in determining the electrochemical response of the system [2,3]. One way to modify such systems is to use carbon nanomaterials and enzymes. Carbon nanotubes (CNTs) encompass all the requirements for fabricating an electrode surface with unique electrical, chemical, and mechanical properties which can be further modified to enable the detection of biomolecules. CNTs are an allotrope of graphitic carbon in the nanometre scale, which consists of one or more concentric tubules, each with a helically wound hexagonal honeycomb lattice structure [4]. CNTs can be single walled or multiwalled in accordance with the number of atomic layers in the CNT walls [5]. Multiwall carbon nanotubes (MWCNT) consist of multiple layers of concentric single-walled graphene cylinders with an interlayer spacing of 3.4 Å, held together via Van der Waals forces [6]. They have major advantages over their single-walled counterparts, for biosensing applications in particular, such as an increased surface area, enhanced electron transfer rates between active centres of biomolecules and the electrode, increased stability, and multiple acidic sites which enable the high loading capacity of proteins (such as enzymes) [7,8]. Electrodes which are functionalised with MWCNTs have demonstrated their advantages for being utilised in sensing devices [9].

The use of MWCNTs to modify electrode surfaces to increase their potential application as biosensors has been previously explored [10]. However, the pre-processing of carbon materials remains a significant barrier in the exploitation of their unique properties. Due to their insoluble nature, MWCNTs are difficult to effectively disperse in solution without the use of mechanical stimulation or the use of harsh solvents, thus measures must be taken to facilitate more efficient dispersion [11]. MWCNTs have previously been shown to achieve dispersion through non-covalent interactions with surfactants and polymers with functional groups that assemble onto MWCNT surfaces via π-π interactions [12].

Different methods of biomolecule immobilisation have been studied using either physical or covalent methods [10,13]. Such immobilisation techniques can further enhance the enzymes’ catalytic stability, reusability, and specificity depending on the method used. However, whilst non-covalent methods of biomolecule immobilisation are frequently simple and utilised, covalent methods using crosslinker molecules have been shown to result in significantly increased biomolecule loading and more favourable protein orientation [14]. Direct adsorption is one of the simplest methods of chemical free binding and is cost efficient and rapid to carry out. However, enzyme loading is of concern with this technique due to inefficient immobilisation resulting in inadequate electron transfer rates. On the other hand, covalent binding has been one of the most studied methods of protein immobilisation on surfaces. Biomolecules such as enzymes bound via covalent attachment produce more stable immobilised enzyme-nanotube preparations, in comparison to physical adsorption. MWCNTs can be treated with oxidising agents to induce functional groups onto the sidewalls of the nanotubes [15]. These new functional groups can interact with other reactants to alter the solubility of MWCNTs in water and other organic solvents. By controlling this process MWCNTs can be exploited as transducers for the catalytic detection of biomolecules, since the enzymes directly bind to the MWCNTs without their activity being compromised.

One of the major advantages for electrochemical biosensing platforms is that MWCNTs can serve as scaffolds for the immobilisation of biomolecules onto their surface [13]. The immobilisation of biomolecules such as antibodies, enzymes, and nucleic acids onto electrodes allow for the development of highly sensitive, and disposable devices due to increased substrate specificity [16]. Enzymes are fragile biomolecules and are prone to denaturation in conditions of organic solvents, pH, and temperature outside their optimum requirements [17,18]. Thus, to improve their stability, enzymes have been frequently immobilised onto a solid carrier such as MWCNTs. Various nanocomposites have been employed in the immobilisation and stabilisation of enzymes [19]. The architecture of the nanotube scaffolds has an inherently large surface area which subsequently leads to high enzyme loading and an increase in enzyme volumetric activity [14].

In this study, the enzyme diamine oxidase was selected to act as a transduction element for the potential application of biomolecule sensing. Diamine oxidase is an enzyme which interacts with the metabolite cadaverine, a major exacerbation component in diseases such as periodontal disease and chronic kidney disease to which this device may demonstrate applicability within the healthcare field [20].

This study was conducted to demonstrate the homogenous dispersion of MWCNTs in solution via the use of the enzyme diamine oxidase to develop a more homogenous electrode surface, with a larger surface area to volume ratio for applications in biological biosensing.

## 2. Materials and Methods

The chemicals utilised in this study were of analytical grade or higher and were purchased directly from Merck (formerly Sigma-Aldrich), Gillingham, UK, unless stated otherwise. All solutions were prepared with Type-1 Milli-Q water with a resistivity no less than 18.2 MΩ cm and degassed vigorously using nitrogen prior to any electrochemical measurements in order to remove any residual oxygen species.

### 2.1. Fabrication of Screen-Printed Electrodes

The in-house fabricated screen-printed electrodes (SPEs) were printed with stencil designs to achieve a 3 mm diameter working carbon electrode, using carbon graphitic ink (Gwent Electronic Materials Ltd., Pontypool, UK). A microDEK 1760RS screen-printing machine (DEK, Weymouth, UK) was used to print a carbon graphitic ink formulation (product code C2000802P2: Gwent Electronic Materials Ltd., Pontypool, UK) onto a flexible polyester film substrate (Autostat, Milan, Italy). The layer of carbon graphitic ink was cured in a fan oven at 60 °C for 30 min. An Ag/AgCl reference electrode was added via the screen-printing of an Ag/AgCl paste (product code C2040308D2: Gwent Electronic Materials Ldt, Pontypool, UK) onto the polyester substrate. After curing at 60 °C for a further 30 min, a dielectric paste (product code D2070423D5: Gwent Electronic Materials Ltd., Pontypool, UK) was printed onto the polyester flexible film to cover and secure the connections. After a final curing process at 60 °C for 30 min, the SPEs were stored at room temperature until ready for use.

### 2.2. MWCNT Suspensions

Individual carbon nanotube suspensions were prepared in 1 mL aliquots in order to draw appropriate comparisons. MWCNTs were placed in a low retention Eppendorf tube (Eppendorf, Stevenage, UK) to minimise carbon complex formations. Two milligrams of MWCNT were added to each of 10 mL deionised H_2_O (dH_2_O), 100% ethanol, 2-(N-morpholino)ethanesulfonic acid (MES), and diamine oxidase (DAO) solution in phosphate buffer (enzymatic solution) and ultra-sonicated at 42 KHz for 20 min to evenly disperse the MWCNTs.

### 2.3. Carboxylation of MWCNTs

An acidic solution containing 7.5 mL H_2_SO_4_ (98%) and 2.5 mL HNO_3_ (70%) was used to incorporate carboxyl groups onto the MWCNTs outer surfaces. For complete carboxylation, 2 mg of MWCNTs were sonicated in acidic solution for 6 h at 80 °C. After completion of the acid etching, the carboxylated MWCNTs (C-MWCNTs) were washed with dH_2_O and vacuum filtered until a pH of 7.2 was achieved to ensure removal of any residual acids and were dried in an oven at 90 °C overnight.

### 2.4. EDC-NHS Coupling of C-MWCNTs

To couple the DAO enzyme to the C-MWCNTs, a two-step procedure was undertaken. Two milligrams of C-MWCNTs were suspended in 2 mL of MES buffer and sonicated for 10 min, followed by elution in 1.2 mL of a 10 mg mL^−1^ 1-ethyl-3-(3-dimethylaminopropyl)carbodiimide hydrochloride (EDC) solution (pH 6.5) for 30 min at room temperature. Next, 2.2 mL of a 50 mg mL^−1^ *N*-Hydroxysuccinimide (NHS) solution was added to the C-MWCNT/EDC solution and incubated at 37 °C for 30 min under continual stirring to allow for completion of the EDC-NHS coupling reaction. The C-MWCNT/EDC-NHS solution was filtered through a 0.45 µm PTFE membrane filter and rinsed using 5 mL of 50 mM MES buffer solution (pH 6.5) to remove any unconjugated EDC, NHS, or cross-linked urea. The C-MWCNT/EDC-NHS were dried in a class II cabinet for one hour before being stored at 4 °C until required.

### 2.5. Preparation of DAO-Conjugated C-MWCNT/EDC-NHS

Two milligrams of C-MWCNT/EDC-NHS in 2 mL of MES (50 mM at pH 6.5) were added into 1 mL of DAO solution (10 mg mL DAO in 0.1 M phosphate buffer) and the mixture was incubated at 37 °C for 1 h under constant shaking (200 rpm) for covalent conjugation of the enzyme to develop with the amine group complexes. Crosslinking was performed via the addition of 1 mL of a 0.2% glutaraldehyde (GA) solution to the C-MWCNT/EDC-NHS/DAO solution, and the suspension was incubated at room temperature under constant shaking for 30 min, followed by an overnight incubation at 4 °C. After the overnight incubation, the C-MWCNT/EDC-NHS/DAO/GA suspension was treated with Tris buffer (100 mM at pH 7.2) for 30 min, and washed to remove unconjugated enzyme and GA. The crosslinked C-MWCNT/EDC-NHS/DAO/GA remained suspended in 0.1 M MES buffer and stored at 4 °C until ready to use (Figure S1 Supplementary Information).

### 2.6. C-MWCNT/EDC-NHS/DAO/GA Electrode Modification

The working electrode of the SPE was prepared for modification via C-MWCNT/EDC-NHS/DAO/GA by initially rinsing with 2 mL of H_2_O. Three replicate SPEs were attached to a Petri dish using 10 mm × 10 mm squares of double-sided tape and placed in a class II cabinet to eliminate any potential contaminants to the working electrodes. The C-MWCNT/EDC-NHS/DAO/GA solution was sonicated for 10 min to homogenise the suspension and a 10 µL volume was drop cast onto the working electrode, ensuring the SPEs remain completely unagitated. The modified SPEs were dried in the class II cabinet for 1 h. The C-MWCNT/EDC-NHS/DAO/GA SPEs were stored in individual sterile plastic 5mL bijous with 1 mL MES buffer at 4 °C solution until ready for use.

### 2.7. Surface Roughness Measurements 

Optical profilometry was used to obtain the surface topography (roughness parameters) of the carbon electrode surfaces pre- and post-MWCNT conjugation. Analysis of the surface roughness was carried out qualitatively via images and quantitatively by calculating *S* values, *S_a_*, *S_q_* and *S_pv_* (arithmetical mean height, mean square roughness, and mean square height, respectively). The average peak and valley height and widths were also measured (*n* = 3).

### 2.8. Water Contact Angle Measurements 

Contact angle measurements of the carbon electrode surfaces pre- and post-C-MWCNT/EDC-NHS/DAO/GA modification were determined at room temperate using the sessile drop technique [21]. HPLC grade water (VWR Chemicals BDH, Lutterworth, UK) at a droplet size of 5 µL was deposited onto a horizontally working electrode, and measurements were recorded using a goniometer with surface contact angle images being analysed using Krüss SW23 (DSA2) (Krüss, Hamburg, Germany) software (*n* = 3).

### 2.9. Scanning Electron Microscopy (SEM) and Energy Dispersive X-ray Spectroscopy (EDX)

MWCNT suspensions (unmodified MWCNT and C-MWCNT/EDC-NHS/DAO/GA) were prepared by pipetting 10 µL of the sample solution onto 10 mm × 10 mm silicon wafer squares (Montco Silicon Technologies Inc., Spring City, PA, USA) and dried in a class II cabinet for 1 h. The dried samples were submerged in 4% *v*/*v* GA overnight, followed by dehydration of the MWCNT via an ethanol gradient series of 30%, 50%, 70%, 90% and 100% *v*/*v*, respectively, for 10 min. The electrodes were mounted on aluminium SEM mounts (Agar, Scientific, UK) with double-sided conducting carbon tabs (Agar Scientific, Stansted, UK). Prior to imaging, the electrode surfaces were sputter coated with gold-palladium and imaged using a Zeiss Supra 40VP scanning electron microscope (Zeiss, Cambridge, UK) utilising the following parameters: acceleration voltage, 2.00 kV; working distance, 4.1–4.5 mm; SE2 detector, magnification at 10,000×. EDX analysis was also carried out alongside the SEM and was utilised to determine the chemical composition of the carbon nanotube modifications. Measurements were made using an EDX Sapphire Si (Li) detector and quantified using a standardless ZAF algorithm. The chemical composition was calculated as an atomic percentage, giving the percentage of the atom relative to the total number of atoms per scan (*n* = 3).

### 2.10. UV-Vis Spectroscopy

Bulk MWCNTs and C-MWCNTs were prepared in 2 mg aliquots and dispersed in 2 mL of 100% ethanol prior to UV-Vis analysis. A high precision quartz glass cuvette (Hellma Analyticis, Southend on Sea, UK) was used for all absorbance readings and 2 mL of 100% ethanol was used for calibrations. For the UV-Vis analysis of the MWCNT, a Thermo scientific Evolution™ 201 UV-Visible spectrophotometer was used, and spectra were recorded using the INSIGHT™ software at the 1000 nm to 200 nm range (*n* = 3).

### 2.11. Raman Spectroscopy

Raman spectroscopy was performed on the MWCNT modifications using a DXR Raman microscope (Thermo Scientific, Newport, UK) fitted with a 532 nm excitation laser at a low power of 3 mW to avoid heating effects. Spectra were recorded using a three-second exposure time for three accumulations at each point (*n* = 6).

### 2.12. Electrochemical Measurements 

Electrochemical analysis of the modified carbon electrodes was carried out using an EmStat2 (Palmsens, Houten, The Netherlands) potentiostat, utilising the PStrace (version 5.8) software (Palmsens, Houten, The Netherlands). Measurements were taken using a three-electrode system, with a platinum wire counter electrode and an Ag/AgCl reference electrode with the screen-printed modified carbon working electrode, completing the circuit. All measurements were made at room temperature in a cell consisting of a supporting electrolyte solution of 0.1 M potassium chloride (KCL) at pH 7.2 with continuous stirring.

### 2.13. Electrochemical Characterisation of the Modified SPEs

The electrodes were characterised using cyclic voltammetry with the outer sphere redox probe, [Ru(NH_3_)_6_]^3+/2+^ at a concentration of 1 mM in 0.1 M KCL. The following scan rates were used: 5 mV s^−1^, 10 mV s^−1^, 15 mV s^−1^, 25 mV s^−1^, 50 mV s^−1^, 75 mV s^−1^, 100 mV s^−1^, 150 mV s^−1^, 250 mV s^−1^, and 500 mV s^−1^. The electrochemically effective area (*A*_eff_) of the modified electrode was determined using the Randles-Ševčík equation for an electrochemically quasi-reversible process [22]:

(1) $$I_{p,f}^{quasi}=\pm 0.436\;nFA_{real}C\sqrt{\;\frac{nFDv}{RT}}$$

where *I_p,f_* is the voltammetric peak current (analytical signal) determined using the forward peak for a quasi-reversable process, *F* is the Faraday constant, *C* is the concentration of the redox-probe under investigation in mol^−1^, *n* is the number of electrons per molecule involved, *D* is the diffusion coefficient in cm^2^ s^−1^, *v* is the voltammetric scan rate in V s^−1^, *R* is the universal gas constant, *T* is the temperature in *K*, and *A_real_* is the electroactive area of the electrode in cm^2^.

The heterogenous electron transfer (HET) rate constant (*k*^0^) was calculated using the Nicholson equation for an electrochemically quasi-reversable process:

(2) $$\varphi =k^{0}\;{\left[\left(\pi DnvF\right)/\left(RT\right)\right]}^{-1/2}$$

where φ is the kinetic parameter which is represented as a function of peak-to-peak separation (Δ*E*_p_) at a temperature of 298 K for a one-step, one-electron process. The function of Δ*E*_p_ which fits the equation for practical purposes was used [23,24]. Thus, making the evaluation of the rate constant simpler and extending the Nicholson parameter towards significantly higher/lower peak potentials:

(3) φ=(−0.6288+0.0021X)/(1−0.017X)

where *X* is equal to Δ*E*_p_ and is used to determine φ as a function of Δ*E*_p_ from the experimentally measured voltammogram. As a result, *φ* against [(*πDnvF*)/(*RT*)]^−1/2^ can be plotted, thus enabling the determination of *k*^0^ from the gradient. The *k*^0^ values were calculated assuming the diffusion coefficient for hexaammineruthenium(III) chloride was 9.10 × 10^−6^ cm^2^ s^−1^.

### 2.14. Statistical Analysis

Statistical analysis of the results was carried out using GraphPad Prism 9 and unpaired *t*-tests or one-way/two-way ANOVA comparison tests were opted for use. In each instance a *p* < 0.05 was deemed statistically significant.

## 3. Results

**Figure 1:**
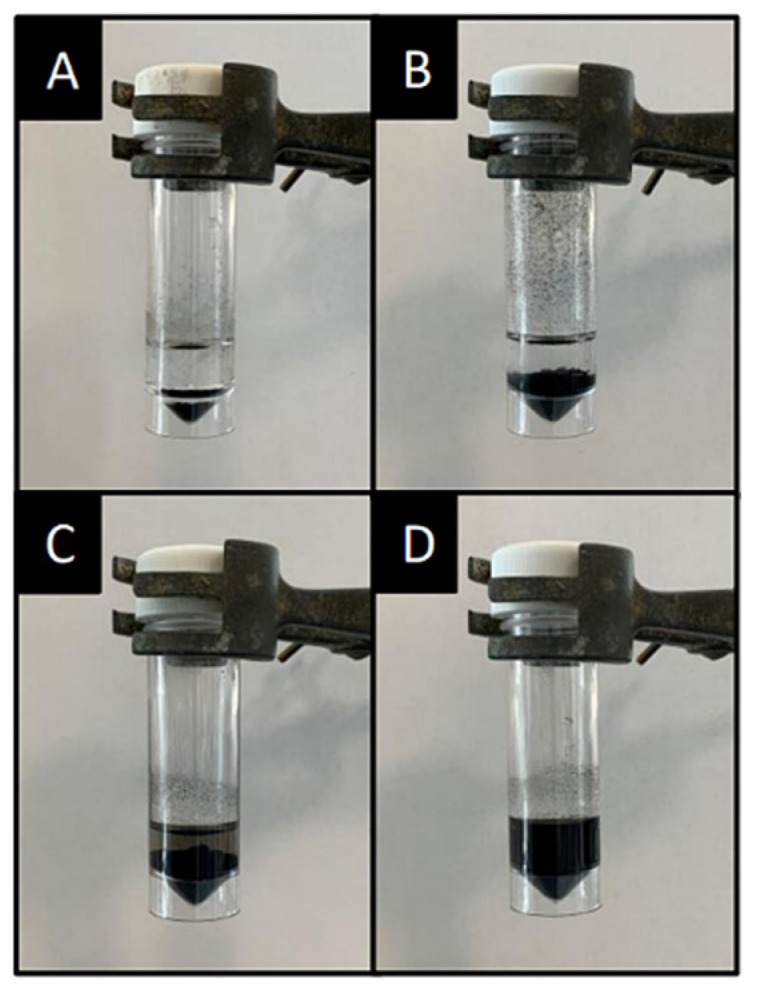
MWCNT suspensions in 10 mL of (**A**) dH_2_0, (**B**) 100% ethanol, (**C**) MES buffer, and (**D**) diamine oxidase enzymatic solution after ultrasonication for 20 min and left undisturbed for 2 h.

Due to the hydrophobic nature of carbon nanotubes, which quickly form aggregates when exposed to water, they required a pre-functionalisation step or the use of solvents to homogenise them in suspension. The initial dispersibility of MWCNTs in dH_2_O (Figure 1A, 100% ethanol (Figure 1B), MES buffer (Figure 1C), and an enzymatic solution consisting of 0.5 U mL of DAO in 0.1 M phosphate buffer (pH 7.2) (Figure 1D) was determined. The MWCNTs achieved the most homogeneous dispersion when solubilised in an enzymatic solution (Figure 1D) and remained evenly dispersed over 24 h. Similarly, the use of MES (Figure 1C) resulted in effective initial MWCNT dispersibility, however, once left unagitated overnight, the MWCNTs reformed aggregates and congregated at the bottom of the universal. The MWCNTs suspended in dH_2_O and 100% ethanol solutions initially formed a homogeneous solution under ultrasonication, however, both solutions aggregated immediately and separated from the solution after ceasing sonication and within 2 h of being left undisturbed.

**Figure 2:**
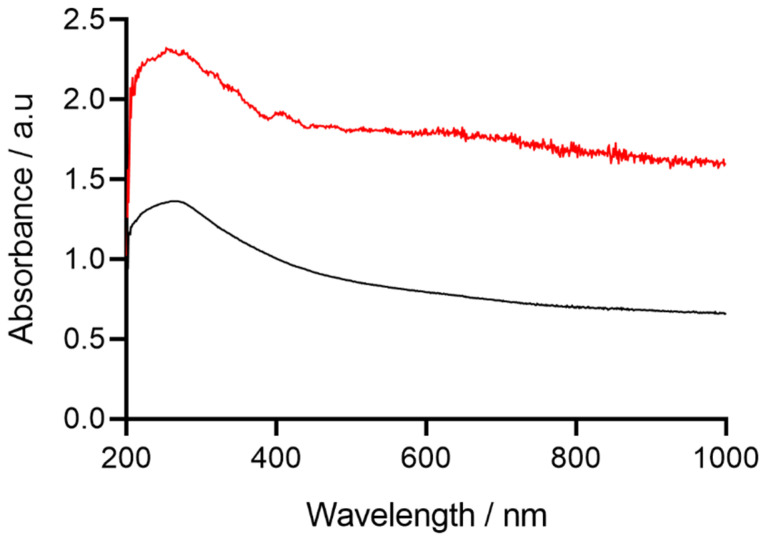
UV-Vis absorption spectra demonstrating the peak intensity shift of MWCNTs (Black) and carboxylated MWCNTs (Red), measured in 100% ethanol.

UV-Vis spectroscopy was utilised to distinguish between MWCNTs in their bulk and carboxylated forms. The spectra demonstrated absorbance peaks at 240 nm for MWNCTs, and at 265 nm for C-MWCNTs. An increase in peak intensity in the absorbance spectra was observed with the C-MWCNTs due to the increase in the percentage surface oxygen, which was indicative of nanotube carboxylation. The increase in the degree of absorbance reflected the increase in oxygenation on the surface of the MWCNTs determined from the spectra (Figure 2) and enabled the differentiation of MWCNTs from C-MWCNT variants.

**Figure 3:**
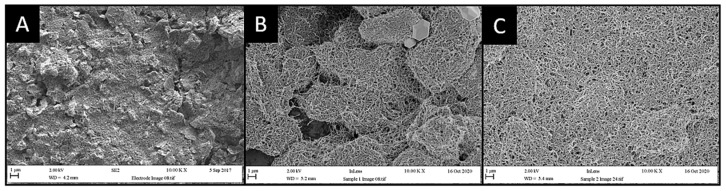
SEM micrographs of MWCNTs in sonicated solutions of (**A**) dH_2_O, (**B**) enzymatic solution, and (**C**) MES buffer solution. Typical heterogeneous surfaces are demonstrated by carbon SPEs (**A**) contributing to MWCNT aggregate formation (**B**). Under enzymatic interaction, MWCNTs demonstrate more homogeneous dispersion with the electrode surface (**C**).

To evaluate the morphology of the electrode surface, SEM micrographs of the MWCNTs were used to verify and evaluate the changes the nanotubes had made on the SPE surfaces. SEM images (Figure 3) were taken of an unmodified electrode surface (Figure 3A), non-functionalised MWCNT on the electrode surface (Figure 3B), and MWCNTs with complete enzyme crosslinking (Figure 3C). The bare electrode surface (Figure 3A) demonstrated an uneven heterogeneous surface which is typical of carbon screen-printed electrodes. Due to irregularities on the SPE surface, the addition of non-functionalised MWCNTs resulted in the formation of irregular agglomerates. However, the enzyme crosslinked with MWCNTs (Figure 3C) demonstrated the opposite effect on the electrode surface, showing more homogeneous distribution.

**Figure 4:**
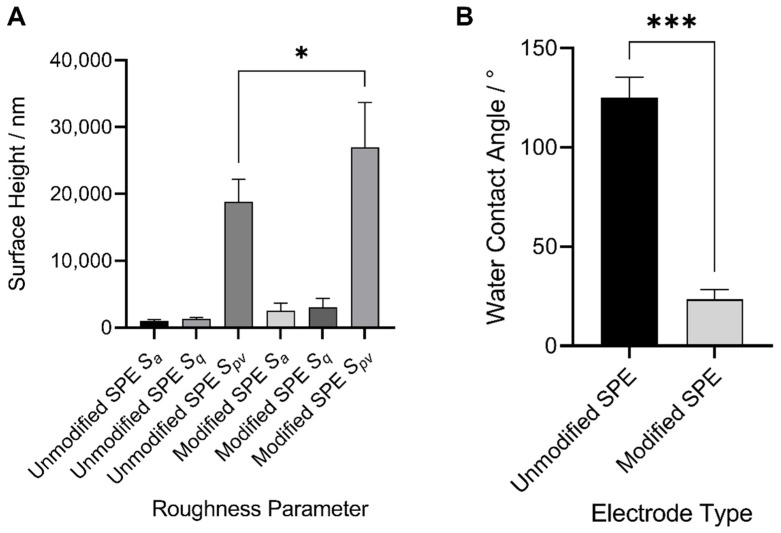
(**A**) Surface roughness measurements (*S_a_*, *S_q_* and *S_pv_*) of carbon screen-printed electrodes after drop casting of MWCNT formulation. *S* values were determined using optical profilometry. (**B**) Water contact angles of the unmodified carbon screen-printed electrode and after the deposition of the MWCNT formulation. * and *** indicates a *p* value of <0.05 and <0.0001 respectively.

Surface roughness measurements were conducted using optical profilometry in order to evaluate the degree of roughness which was influenced by the deposition of the functionalised MWCNTs onto the electrode surfaces (Figure 4A). The *S_a_* values demonstrated the arithmetical mean height of the surface. The pre- and post-modified surfaces showed an increase in overall surface roughness. Similarly, the mean square roughness of the surface (*S_q_*) demonstrated an increase in roughness on the SPE after the addition of the MWCNT formulation, but again this was not found to be significantly different. However, the mean maximum height of the surface (*S_pv_*) showed a significant increase after the application of the MWCNT on to the SPEs due to MWCNT aggregate formation on the upper ridges of the carbon SPE. 

In this study, water contact angle measurements were carried out to determine wettability changes with the addition of the MWCNTs on the carbon electrode surface (Figure 4B). The initial unmodified SPE demonstrated a water contact angle of 125.0° which subsequently decreased significantly with the deposition of the MWCNT formulation, resulting in a water contact angle of 23.5° and a more wettable and hydrophilic electrode surface. 

**Figure 5:**
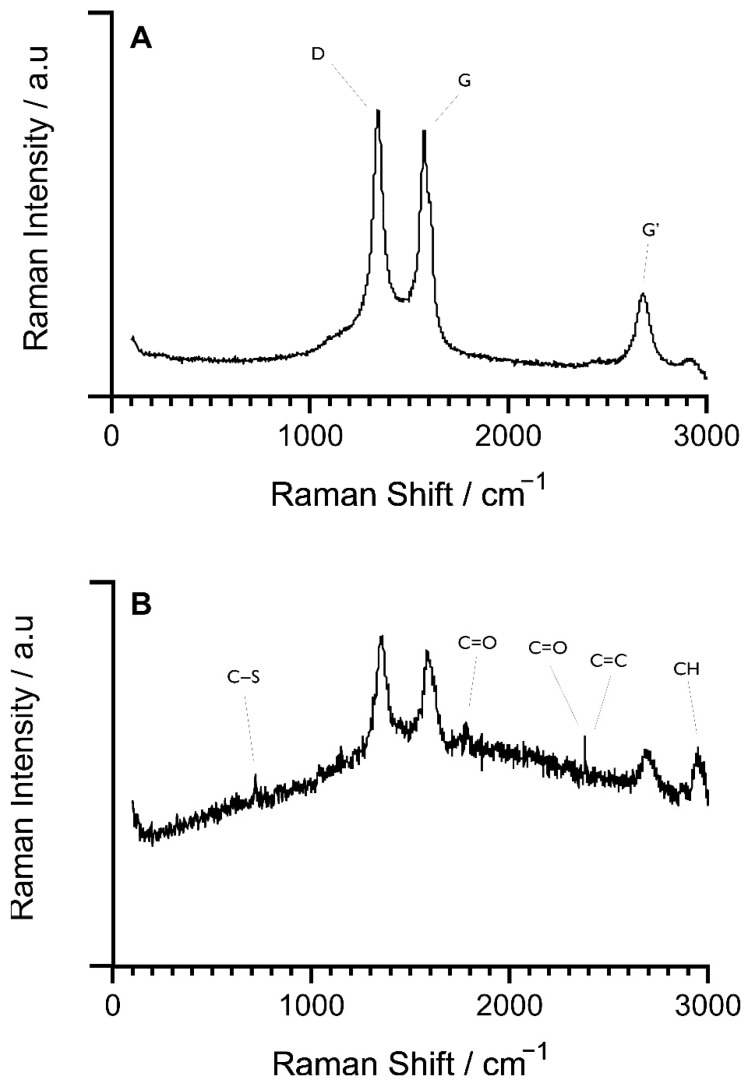
Raman spectroscopy of (**A**) bulk MWCNTs and (**B**) functionalised MWCNTs demonstrating the D (1350 cm^−1^), G (1560 cm^−1^), and G’ (2700 cm^−1^) vibrational peaks representative of sp^2^ carbon nanomaterials. Further peaks at 719 cm^−1^, 1783 cm^−1^, 2382 cm^−1^, 2387 cm^−1^, and 2947 cm^−1^ were identified to be indicative of the MWNCT diamine oxidase functionalisation.

Raman spectroscopy was used to determine the spectral fingerprint of the unmodified and modified SPE surfaces. The Raman spectra of bulk MWCNT (Figure 5A) and enzyme-functionalised MWCNTs was determined (Figure 5B). For both spectra, the radial breathing mode (RBM) was not present (100–200 cm^−1^), which is typical of multiwalled nanotube variants of carbon nanomaterials. The G band (identifiable at the 1560 cm^−1^ position) demonstrated the stretching mode of the C-C bonds which form the hexagonal lattice structure of all sp^2^ carbon nanomaterials. Due to the presence of defects on MWCNT surfaces, a D band (1350 cm^−1^) was present on hexagonal sp^2^ materials, with both unmodified and modified samples demonstrating these peaks. Furthermore, at the 2700 cm^−1^ wavelength, the vibrational mode characterised by the breathing of six carbon atoms in a hexagonal lattice structure of graphene derivative molecules known as the G’ (2D) band was observed.

The functionalised MWCNTs demonstrated (Figure 5B) discrete Raman peaks in addition to those presented by unmodified MWCNTs. Peaks were measured at 2387 cm^−1^ which can be related to the D + D″ band introduced by the presence of defective graphitic carbon. Furthermore, peaks at 2947 cm^−1^ were observed and denote C-H stretching modes. At lower frequencies, vibrational peaks at 2382 cm^−1^ and 1783 cm^−1^ were observed and demonstrated C=C and C=O stretching bonds, respectively. Finally, a protein identification vibrational mode at 719 cm^−1^ was determined as C-S bonds. From the generated spectra it was possible to determine the different vibrational modes of each MWCNT sample.

EDX analysis was used to identity the elemental composition of the MWCNTs in bulk form and after enzyme conjugation (Table 1). The presence of carbon, oxygen, sodium, phosphorus, and sulphur was demonstrated. The addition of nitrogen, silicon, and chlorine were observed in the MWCNT formulation. The presence of each element was quantified from the EDX data, and as expected in both samples, carbon was the most abundant element, followed by oxygen. Differences could be observed in the pre- and post-conjugation samples due to the presence of EDC and NHS compounds and enzymes, as demonstrated by the additional presence of nitrogen, silicon, and chlorine groups. Furthermore, decreases on both weight percentage and atomic weight in the post-modified samples were observed due to the increasing number of molecules per element present in each individual functionalisation stage.

**Figure 6:**
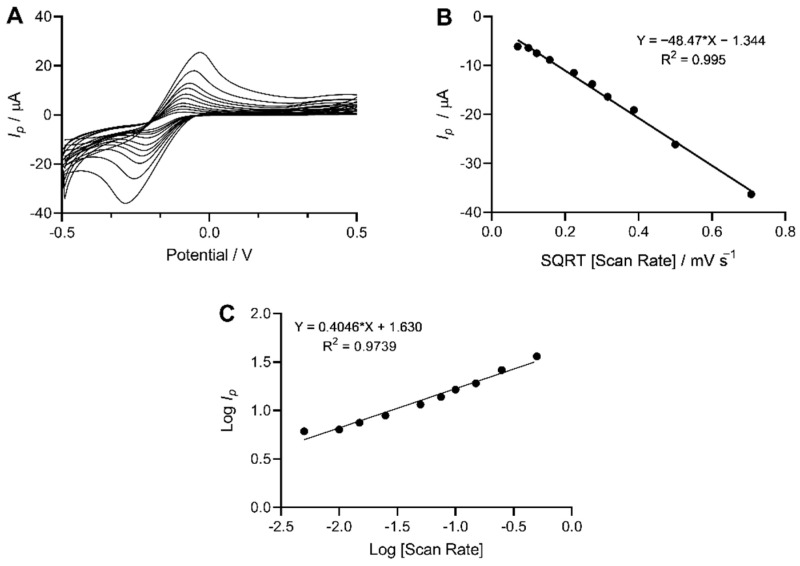
Electrochemically effective area of MWCNT-functionalised SPE determined using 1 mM hexaammineruthenium(III) chloride within a potential window of −500 mV to + 500 mV s^−1^ in 0.1 M KCL vs. Ag/AgCl reference electrode. (**A**) Cyclic voltammograms were recorded at increasing scan rates of 5 mV s^−1^, 10 mV s^−1^, 15 mV s^−1^, 25 mV s^−1^, 50 mV s^−1^, 75 mV s^−1^, 100 mV s^−1^, 150 mV s^−1^, 250 mV s^−1^, and 500 mV s^−1^ (increasing scan rate corresponds to increasing distance between forward and reverse scans). (**B**) Linearity of peak current vs square root of the scan rate with R^2^ of 0.995. (**C**) Log of peak current as a function of log scan rate used to determine gradient for a diffusion controlled electrochemical process.

The voltametric response of the MWCNT-modified SPEs was evaluated electrochemically using the outer sphere redox probe, hexaammineruthenium(III) chloride. The observed electrochemical behaviour of the redox probe recorded using the MWCNT modified SPEs was demonstrated (Figure 6). The voltametric peak-to-peak potential (Δ*E*_p_) of the oxidation and reduction process of the redox couple was evaluated. The heterogenous electron transfer rate was calculated for the MWCNT electrode and resulted in a *k*^0^ value of 1.71 × 10^−3^ cm s^−1^, an *A*_eff_ of 0.0603 cm^2^, and *A*_real_ of 87.70%. The Δ*E*_p_ for the MWCNT-functionalised SPE was 150 mV s^−1^ at 100 mV s^−1^. Furthermore, peak current was plotted vs square root of the scan rate to determine the linearity of the plot and results demonstrated a linear relationship with an R^2^ = 0.995. The log of the peak current was also plotted against the log of scan rate to determine the gradient. The gradient was calculated to be 0.4, which is near the theoretical 0.5 for a diffusion-controlled process.

## 4. Discussion

### 4.1. MWCNT Dispersibility

MWCNT suspensions are one method in which nanomaterials may be utilised to modify electrodes for biosensor applications. Due to their biocompatibility, MWCNTs are ideal candidates for biomolecule loading, thus significant research is being conducted to advance these models, however, major challenges in the way of nanotube pre-processing remain. The formation of MWCNT agglomerations have been suggested to occur due to the hydrophobicity of the sp^2^ carbon sidewalls and the strong π–π stacking interactions between individual carbon nanotubes [25,26]. The surface of unmodified MWCNTs result in a shortage of hydrogen bonding with water molecules. This results in a failure to facilitate stable MWCNT suspensions. This finding is in agreement with Alnarabiji et al. (2016) who used the sessile drop technique directly onto the surface of the MWCNTs and demonstrated a water contact angle of 136°, indicating a hydrophobic structure [27]. It has been suggested that the addition of the enzyme DAO can result in improved carbon nanotube dispersion, hence the rationale for its selection for use in this work. Kim et al. (2017) suggested the amphiphilic nature of enzymes can be a key contributor in the facilitation of CNT dispersion as the hydrophobic moieties of the enzymes interact with the CNT surface and the hydrophilic enzyme residues interact with the dispersal solution, thus inhibiting nanotube aggregation and resulting in a homogeneous CNT suspension [28]. In agreement with this, the work presented herein demonstrated that when the enzyme DAO was used for the facilitation of the dispersion of MWCNTs, a more uniform drop cast on the electrode surface was observed.

### 4.2. UV-VIS

The UV-Vis spectra of the MWCNTs and the C-MWCNTs demonstrated two main absorption features. The π–plasmon absorption peaks at *ca*. 240 nm result from the excitation of the π–electron systems which are apparent in all sp^2^ hybridised carbon materials [29]. In agreement with the results presented in this study, the relative increase in peak absorbance of the C-MWNCT in comparison to the MWCNTs was also determined in other work [30,31]. It was suggested that the increase in absorbance for the C-MWCNTs occurred due to the π–plasmon bands resonating free electrons in the C-MWCNT structures.

### 4.3. Scanning Electron Microscopy

Carbon screen-printed electrodes demonstrate ‘flake-like’ morphologies under SEM imaging. These flakes, which were embedded in the carbon graphitic ink used in their production, have been evaluated previously [32]. The characteristic cohesion and flake-like structure typical of MWCNT behaviour were observed on the electrode surfaces which has been previously shown to be due to the van der Waals interactions between each nanotube [33]. However, after MWCNT functionalisation, the arrangement of MWCNT structures on the electrode surfaces decreased the amount of aggregation present and resulted in an improved MWCNT arrangement due to the hydrophobic interactions between the nanotubes [34]. The resultant ‘thread-like’ woven mesh morphology arises due to functionalisation steps taken on MWCNTs and resulted in less aggregated, more homogeneous complexes after drop casting on the electrodes, and this has been shown to contribute towards a larger effective electrochemical surface area [35,36,37].

### 4.4. Surface Roughness and Wettability

Screen-printed electrode surfaces have irregular features due to the presence of peaks and valleys of graphitic carbon originating from the initial printing process. The surface profiles of the unmodified electrodes were measured to determine if the roughness of the surface changed with the deposition of the MWCNT formulation. The unmodified carbon working electrodes initially presented with a less rough surface, which could be attributed to the binder utilised in their production filling in the surface features [38]. In contrast, the MWCNT functionalised electrodes demonstrated significantly increased roughness profiles due to the formation of MWCNT aggregates upon interaction with the carbon substrate. MWCNTs have been shown to accumulate on surface peaks and features, thus exaggerating surface peak height and increasing overall roughness profiles. An increase in electrode roughness as a result of carbon nanotube deposition was similarly observed by Ziyatdinova et al. (2012) where the modification of glassy carbon electrodes led to a significant increase in surface roughness, which was again suggested to be due to the formation of CNT aggregates on the electrode surface [39]. Sun et al. (2015) demonstrated that the application of CNTs to a graphene surface not only increased the surfaces overall roughness but also improved the electrical conductivity between the surface and the CNTs via the introduction of a greater number of enzyme active sites for increased biocatalytic activity [40,41]. This was particularly important, as this increase in conductivity aided in the measurements of lower analyte concentrations, thus increasing overall device sensitivity [42].

In agreement with the findings presented in this study, an increase in surface wettability have also been demonstrated by the modified electrode surfaces. This has been suggested to be a result of the introduction of more polar head groups of -COOH on the electrode surface, thus presenting as a more hydrophilic surface [37,43]. Previous studies have reported that an increase in the hydrophilicity of electrodes resulted in more efficient analyte detection at the electrode interface [44].

### 4.5. Raman Spectroscopy

Raman spectroscopy is an effective technique in the characterisation of carbon materials due to its non-destructive nature and its sensitivity to structural changes at the molecular level. Raman analysis of MWCNTs within this study was conducted on samples with the same concentration of nanotubes. The predominant features of carbon nanomaterials can be identified by the presence of the G and D peaks which were evident around 1560 cm^−1^ and 1360 cm^−1^, respectively, for visible excitation [45]. Two different formations of nanotubes were evaluated, non-functionalised MWCNT and the C-MWCNT/EDC-NHS/DAO/GA. Raman analysis demonstrated typical spectra for MWCNTs in their bulk form, with D bands (A_1g_ mode) corresponding to the sp^3^ disorder carbon–carbon rings of defective graphene structures, and the G band (E_2g_ mode) characteristic of graphitic layers in the planar sp^2^ bonded stretching configuration carbon [46]. The second harmonic order G’-band (2D band) was also observed within the unmodified MWCNT Raman spectra. The identification of these two bands determined the presence of semiconducting and metallic MWCNTs within the observed samples and is in agreement with previous characterisation of MWCNTs [47,48,49]. C-MWCNT/EDC-NHS/DAO/GA characterisation showed additional Raman peaks that denoted the presence of DAO, EDC–NHS, and GA. The presence of the protein crosslinking reagent GA was observed through the identification of C-S (organosulfur) bonds and C=O which prevail in the GA structure [50,51]. Identification of the enzyme DAO was also determined by the presence of C-H stretching bands at the 2947 cm^−1^ range. Work by Sebek et al. (2011) also determined that within the 2900 cm^−1^ range, C-H stretching bands were dominantly present in aliphatic molecules as proteins, thus confirming the detection of the aliphatic DAO [52]. The structure of the MWNCTs was impacted by the numerous stages of modification, thus the D + D″ band was observed as an indicator of inactive modes of defective graphitic carbon, with the D + D″ band seen as the combination of the inactive D photon within a further inactive D″ mode [53].

### 4.6. Electrochemical Analysis

The electrochemical behaviour of the MWCNT modified electrodes was investigated pre- and post-modification to determine the ability of the MWCNT formulation to relay an electrochemical response. The voltammetric response of the modified SPE was explored via the redox probe, hexaammineruthenium(III) chloride [54]. The redox behaviour of the SPE was recorded as voltammetric peak-to-peak potential (Δ*E*_p_) of the observed oxidation and reduction peaks [55]. The reversible limit, which is at Δ*E*_p_ 59 mV s^−1^ (298 K) and smaller potentials up to this limit, demonstrated a more reversible electrochemical process [56]. The voltammetric analysis of the C-MWCNT/EDC-NHS/DAO/GA SPE profiles demonstrated an Δ*E*_p_ of 150 mV s^−1^. The increase in peak-to-peak separation was potentially due to the percentage of binder used in the electrode’s fabrication, which has been shown to reduce electron transport at the carbon structure [57]. Furthermore, analysis of the SPEs via the deduction of the heterogeneous electron transfer rate constant value, *k*^0^ of hexaammineruthenium(III) chloride for the modified SPE, corresponded to 1.71 × 10^−3^ cm s^−1^. To evaluate the MWCNT structure of the electrode and to confirm its non-porous nature, scan rate studies were carried out where the log of the peak current was measured against the log of the scan rate. For a typical diffusion-controlled redox process at a working electrode, one would expect a gradient of close to theoretical 0.5 [58]. The results of this work demonstrated a gradient of 0.4 which is close to the theoretical value of 0.5, thus indicating an electron process which was diffusional and demonstrated a non-porous electrode structure [59].

## 5. Conclusions

This study demonstrated the successful homogenous dispersion of MWNCTs through the manipulation of the hydrophobic/hydrophilic interactions using diamine oxidase to enable a more uniformed MWCNT suspension. Furthermore, the fabrication of screen-printed carbon electrodes was carried out and its surface properties evaluated, which demonstrated that following the use of the enzyme, a surface with increased wettability and roughness was produced which resulted in an increase in electron transfer. SEM, EDX analysis, and Raman spectroscopy confirmed the presence of the MWCNTs on the electrode surface and demonstrated surface changes with the addition of diamine oxidase and further conjugation molecules. Electrochemical voltammetric analysis of the modified electrode demonstrated efficient electron transfer kinetics and determined the process of electron transfer was diffusion controlled as a result of a non-porous modified working electrode. The biosensing device utilised in this study has the potential to be applied to a range of healthcare-related fields for biomolecule detection where increased sensitivity is paramount.

## Figures and Tables

**Table 1:** Elemental composition EDX analysis of MWCNTs in A) unmodified form dispersed in dH_2_O and B) enzymatic solution after complete conjugation (*n* = 3).

Element	At.% Unmodified MWCNT	At.% Modified MWCNT
C	78.19	63.92
O	18.32	19.78
Na	1.68	2.14
P	0.20	0.18
S	1.71	4.78
N	N/A	6.87
Si	N/A	0.45
Cl	N/A	1.89

## Data Availability

The datasets generated during and/or analysed during the current study are available from the corresponding author upon reasonable request.

## Supplementary Materials

The following supporting information can be downloaded at: https://www.mdpi.com/article/10.3390/s22020675/s1, Figure S1. The covalent coupling of 1-ethyl-3-(3-dimethylaminopropyl)carbodiimide hydrochloride and *N*-Hydroxysuccinimide onto carboxylic acid-etched multiwalled carbon nanotubes. The *N*-Hydroxysuccinimide ester reacted with diamine oxidase to form the enzyme-conjugated MWCNTs.
