# Supplementary material for: Diamine Oxidase-Conjugated Multiwalled Carbon Nanotubes to Facilitate Electrode Surface Homogeneity
Source: Sensors (Basel). 2022 Jan 16;22(2):675. doi: 10.3390/s22020675 (PMC8780216; doi:10.3390/s22020675)
Supplement: Supplementary file 1 [file sensors-22-00675-s001.zip › sensors-1522995-supplementary.pdf]

## Supplementary Information

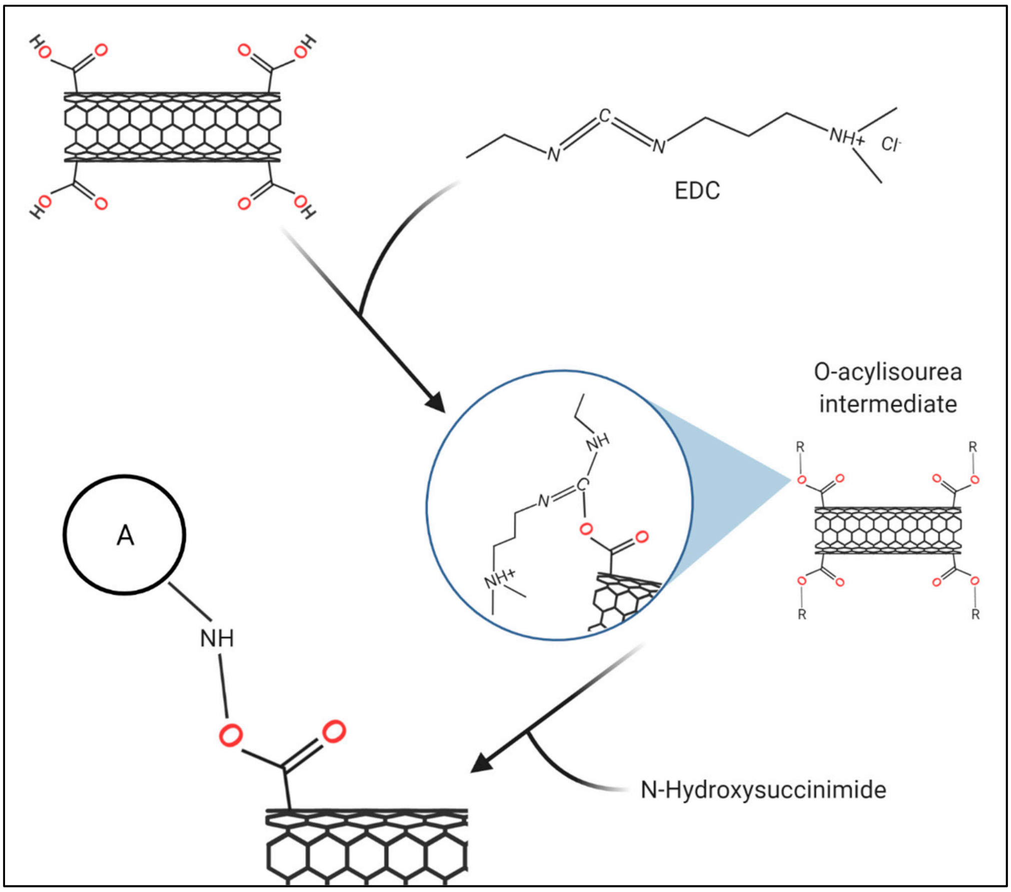


**Figure S1.** The covalent coupling of 1-ethyl-3-(3-dimethylaminopropyl)carbodiimide hydrochloride and *N*-Hydroxysuccinimide onto carboxylic acid-etched multiwalled carbon nanotubes. The *N*-Hydroxysuccinimide ester reacted with diamine oxidase to form the enzyme-conjugated MWCNTs.
